# Investigation of awareness rate of prostate-specific antigen (PSA) among the general public in China and analysis of influencing factors

**DOI:** 10.3389/fpubh.2023.1080800

**Published:** 2023-05-04

**Authors:** Yuqin Wang, Mukun Xiao, Yueying Zhang, Zhiwei Hong, Ruochen Zhang, Qingjiang Xu, Le Lin, Yongbao Wei

**Affiliations:** ^1^School of Clinical Medicine, Fujian Medical University, Fuzhou, China; ^2^Shengli Clinical Medical College of Fujian Medical University, Fuzhou, China; ^3^Department of Urology, Fujian Provincial Hospital, Fuzhou, China

**Keywords:** PSA, prostate cancer, cancer screening, awareness, public, questionnaires

## Abstract

**Objective:**

This study aimed to evaluate the awareness rate of prostate-specific antigen (PSA) among the general public in China and provide data about prostate cancer (PCa) for related scientific research.

**Methods:**

A cross-sectional survey of PSA awareness was conducted in multiple regional populations using an online questionnaire. The questionnaire included basic information, knowledge about PCa, the awareness rate and application of PSA, and future expectations toward applying PSA screening in clinical practice. The study applied the methods of Pearson chi-square analysis and Logistic regression analysis.

**Results:**

A total of 493 valid questionnaires were included. Two hundred and nineteen respondents (44.4%) were males, and 274 (55.6%) were females. Of all respondents, 212 (43.0%) were under 20 years old, 147 (29.8%) were 20–30 years old, 74 (15.0%) were 30–40 years old, and 60 (12.2%) were over 40 years old. There are 310 people (62.9%) with medical educational background and 183 (37.1%) without. One hundred eighty-seven (37.9%) of the respondents were aware of PSA, and 306 (62.1%) were unaware of PSA. Statistically significant differences were obtained between the two groups regarding different ages, educational backgrounds, occupations, departments, and habits of knowing medical knowledge (all *p* < 0.05). In addition, the differences between the group of aware of PSA (AP) and the group unaware of PSA (UAP) in terms of whether they had been exposed to PSA screening and whether they had exposure to PCa patients or related knowledge were also observed (all *p* < 0.05). Age ≥30 years, medical educational background, understanding of medical knowledge, exposure to PCa patients or related knowledge, exposure to PSA screening, and status as a graduate student and above were independent factors for the occurrence of PSA awareness events (all *p* < 0.05). In addition, age ≥ 30 years, medical educational background, and awareness of PSA were independent factors for future expectations toward PSA (all *p* < 0.05).

**Conclusions:**

We first analyzed the public awareness of PSA. Cognition degrees of PSA and PCa awareness vary among different populations in China. Therefore, we should designate corresponding widespread scientific educational programs for different populations to increase the awareness rate of PSA.

## Introduction

Prostate cancer is the second most commonly diagnosed cancer and the fifth leading cause of cancer death among men worldwide, with an estimated 1,414,000 new cancer cases and 375,304 deaths in 2020 (1–3). Although China is still an area with a lower incidence of PCa compared with developed countries such as the United States, the morbidity and mortality of PCa in China have shown a significant rapid increase in the last decade (2, 4, 5). Therefore, it poses a more significant threat to men's health and increases society's economic burden. On the other hand, assuming that PCa could be diagnosed and treated at early stages, the prognosis of the disease will be well-improved (5, 6), and the disability and mortality rates are expected to be reduced.

In recent years, the incidence of PCa has increased significantly while the mortality rate is decreasing, which was associated with the increased usage of prostate tumor markers (PSA) in diagnosing PCa (3, 7, 8). Studies have shown that since prostate-specific antigen (PSA) is applied to clinical practice, the detection rate of localized PCa has increased significantly (9). In contrast, the proportion of high-risk PCa discoveries has decreased. Therefore, it indicates that PSA screening is essential for early detection and diagnosis of PCa, which can reduce the morbidity and mortality of advanced PCa through early treatment. PSA is a chymotrypsin-like acting glycoprotein secreted by prostate columnar epithelium and glandular duct epithelium, which has been widely used for PCa screening (10, 11). In the physiological state, PSA mainly exists in cells and has a lower concentration in the serum. While in pathological states such as PCa and prostatitis, PSA is released from the cell to the serum so that its increasing concentration in the serum can provide a reference for disease diagnosis (6). Studies have shown that the screening and diagnosis efficiency of PCa was optimal when the PSA levels ranged from 4 to 20 ng/mL (5). Generally, the higher the serum concentration of PSA, the more likely prostate cancer is present (6).

A key to improving the early diagnosis of prostate cancer is to increase public awareness of PSA. Elevated awareness of PSA will promote public understanding of the significance of PSA screening and prompt them to undergo this test, thus further facilitate the early diagnosis and treatment of cancer. Furthermore, the prerequisite of popularizing PSA screening is elevating the cognitive level of PSA among the general public. The science popularization toward PSA screening can be carried out on this basis to promote the development of clinical prevention of PCa. Therefore, the survey aimed to assess the awareness of PSA, future expectations, and the factors impacting people's awareness rate of PSA among the general public in China through the form of a questionnaire and provide a scientific basis for the promotion of PSA screening and reference for the popularization of prevention and treatment of PCa.

## Materials and methods

### Respondents

An online questionnaire survey was randomly conducted among people of different ages in multiple regions of China, covering people in various fields to ensure the diversification of respondents. Nationwide, respondents who consented to participate were included in the study. The respondents were from the following geographical regions: Fujian, Liaoning, Shandong, Beijing, Shanghai, Jiangxi, Anhui, Hubei, Hong Kong, Jilin, Jiangsu, Gansu, Hunan, Hebei, Yunnan, Shanxi, Guizhou, Guangdong, Tianjin, Heilongjiang, Sichuan, and so on, covering both rural and urban areas of multiple provinces with a high prevalence of PCa nationwide. Studies have shown that the incidence of PCa in rural areas is higher than in urban areas (12). Therefore, we also included respondents from rural areas of the provinces in our study. In summary, it shows that the valid information obtained from the questionnaire in this study is representative, scientific, and national.

### Questionnaire

A cross-sectional survey design was conducted in this study. According to whether the educational background is relevant to healthcare, the survey divided the population into three groups: cohorts without medical educational background (C-withoutME), cohorts with a medical educational background but without clinical practice (C-withoutCP), cohorts with medical educational background and clinical practice (C-withCP). In addition, different populations can be divided into the AP group (aware of PSA) and the UAP group (unaware of PSA). Based on the characteristics of various cohorts, three targeted observation contents were designed and integrated into a comprehensive questionnaire suitable for the three groups through WENJUANXIN (https://www.wjx.cn/), an effective online questionnaire instrument (13). There are four parts of the questionnaire: (a) basic information (including gender, age, educational background, region, etc.); (b) information about PCa (including whether respondents have a habit of learning medical knowledge, whether respondents know PCa, whether respondents have been exposed to PCa patients, etc.); (c) awareness of PSA and its application (including whether respondents know PSA and its clinical significance, whether respondents have been exposed to PSA screening, whether respondents understand the significance of PSA, etc.); and (d) respondents' future expectations for the application of PSA screening in clinical practice (including whether respondents recognize the necessity of increasing the PSA screening rate, whether respondents hold the point that it is necessary to popularize the prevention and treatment of PCa and increase the public attention to PSA screening and whether respondents are interesting to learn about the scientific knowledge of PSA, etc.).

The questionnaire was distributed through the WENJUANXIN website. The purpose and significance of this survey were explained to respondents at the beginning of the questionnaire. Respondent's personal information was promised to be retained and used only for this study. Under the premise of guaranteeing the principle of informed consent for respondents, questionnaires were completed and submitted by respondents on the website of WENJUANXIN. This survey strictly adhered to the principle of informed consent. It was performed from March 20, 2022, to May 27, 2022. Each mobile phone ID can be filled in only once, ensuring the non-repeatability of the survey results. Simple commonsense questions were included in the questionnaire to ensure the validity of the information collected. Only responses that correctly answered commonsense questions were valid. The questionnaire took 4–6 min to complete, and only the successfully submitted questionnaires were collected.

### Data analysis

All detailed data were exported through the WENJUANXIN website. Four hundred and ninety-three questionnaires remained after eliminating four invalid questionnaires. The filtered Excel files were imported directly into SPSS 26.0 for data entry. SPSS 26.0 was used to analyze the data and make a general statistical description of the obtained data. Counting data were expressed as a percentage (%), and the chi-square test was performed between groups. A *p*-value < 0.05 was considered significant. A logistic regression analysis was performed to explore the factors impacting people's awareness rate of PSA. The logistic regression analysis results were presented as an odds ratio (OR) and a 95% confidence interval (CI).

## Results

### General information

A total of 497 online questionnaires were returned, of which 493 were valid. Two hundred and nineteen respondents (44.4%) were males, and 274 (55.6%) were females. Of all respondents, 212 (43.0%) were under 20 years old, 147 (29.8%) were 20–30 years old, 74 (15.0%) were 30–40 years old, and 60 (12.2%) were over 40 years old. There are 310 people (62.9%) with medical educational background and 183 (37.1%) without. In addition, 187 people (37.9%) were aware of PSA, and 306 (62.1%) were unaware of PSA.

The chi-square test revealed no statistically significant difference between the AP group and the UAP group in terms of different genders (*p* > 0.05). However, there was a statistically significant difference (*p* < 0.05) between the AP and UAP group in terms of different ages and different educational backgrounds (Table 1). The logistic regression analysis showed that age ≥ 30 years and medical educational background were the independent factors impacting people's awareness rate of PSA. The relations were statistically significant (all *p* < 0.05; Table 1).

**Table 1:** Chi-square test and logistic regression analysis of PSA awareness rate.

**Variables**	**Number**	**Chi-square test**		**Logistic regression analysis**		
		*X* ^2^	***P***	**OR**	**95% CI**	***P***
Gender (male/female)	219/274	3.44	0.06			
Age (< 30/≥30 years old)	359/134	137.35	< 0.001	13.82	8.47–22.56	< 0.001
Educational background (unrelated/related to healthcare)	310/183	121.67	< 0.001	18.47	9.87–34.58	< 0.001

In this study, people who believe that it is necessary to incorporate PSA screening into routine physical examinations of the middle-aged and elderly groups, to carry out universal education on PCa and the knowledge about PSA in public, to improve medical practitioners' attention to PSA, and to learn about PCa and PSA in the later stage were considered to have high expectations for the prospect of PSA, regarded as an acceptance and willingness to learn more about the knowledge of PSA. For all populations, there was no statistically significant difference between the two groups with different PSA future expectations regarding different genders (*p* > 0.05). However, there were statistically significant differences between the two groups with different PSA future expectations in terms of different ages, different educational backgrounds, and different PSA awareness (all *p* < 0.05; Table 2). Furthermore, the logistic regression analysis showed that age ≥ 30, medical educational background, and awareness of PSA were independent factors impacting people's future expectations of PSA, and the relations were statistically significant (all *p* < 0.05).

**Table 2:** Chi-square test and logistic regression analysis of PSA future expectations.

**Variables**	**Number**	**Chi-square test**		**Logistic regression analysis**		
		*X* ^2^	***P***	**OR**	**95% CI**	***P***
Gender (male/female)	219/274	0.67	0.41			
Age (< 30/≥30 years old)	359/134	47.46	< 0.001	4.82	3.01–7.72	< 0.001
Educational background (unrelated/related to healthcare)	310/183	35.56	< 0.001	3.12	2.13–4.56	< 0.001
PSA awareness (unaware/aware)	306/187	58.32	< 0.001	4.64	3.09–6.98	< 0.001

### Cohorts without a medical educational background (C-withoutME)

There were 183 respondents in C-withoutME. Eighty-four (45.9%) respondents were males, and 99 (54.1%) were females. There were 131 people under the age of 20 (71.6%), 41 people aged 20–30 (22.4%), three people aged 30–40 (1.6%), and eight people over 40 years (4.4%). Twelve people (6.6%) had a high school or equivalent education, and 171 (93.4%) had a bachelor's degree or equivalent education. The number of people who knew about PCa was 138 (75.4%), while 45 (24.6%) didn't. Regarding the awareness of PSA, 12 (6.6%) said they were aware of it, and 171 (93.4%) said they were unaware. One hundred people (54.6%) had the habit of understanding medical knowledge in ordinary times, and 83 people (45.4%) didn't. Ten (5.5%) people have been exposed to PCa patients, and 173 (94.5%) have not been exposed.

For C-withoutME, there was no statistically significant difference (*p* > 0.05) between the AP group and the UAP group in terms of different genders, ages, educational degrees, and whether or not they had heard of PCa. However, there were statistically significant differences between the AP group and the UAP group regarding whether or not they had habits of understanding medical knowledge and whether or not they had contact with PCa patients (*p* < 0.05). In addition, the results of logistic regression analysis showed that the habit of understanding medical knowledge and exposure to PCa patients were independent factors impacting the PSA awareness rate among C-withoutME, and the relation was statistically significant (*p* < 0.05; Table 3).

**Table 3:** Chi-square test and logistic regression analysis of PSA awareness in C-withoutME.

**Variables**	**Number**	**Chi-square test**		**Logistic regression analysis**		
		*X* ^2^	***P***	**OR**	**95% CI**	***P***
Gender (male/female)	84/99	0.80	0.37			
Age (< 30/≥30 years old)	172/11	2.58	0.11			
Degree (high school and below/college and above)	12/171	< 0.001	1.00			
Medical knowledge understanding habits (no/yes)	83/100	7.10	< 0.01	10.14	1.28–80.24	< 0.05
Ever heard of prostate cancer (no/yes)	45/138	0.10	0.78			
Ever exposed to a prostate cancer patient (no/yes)	173/10	5.87	< 0.05	7.81	1.73–35.35	< 0.01

### Cohorts with medical educational background (C-withME)

There were 310 respondents in C-withME, including 220 respondents who had yet to engage in healthcare and 90 who had engaged in healthcare. One hundred and thirty-five (43.5%) respondents were males, and 175 (56.5%) were females. One hundred and eighty-seven (60.3%) were under the age of 30, and 123 (39.7%) were over the age of 30. The awareness rate of PSA was 56.4%.

For C-withME, there were statistically significant differences between the AP group and the UAP group in terms of different ages, genders, and educational degrees (*p* < 0.05). In addition, the results of logistic regression analysis showed that males, age ≥ 30 years old, graduate degree or above, and already engaged in healthcare are independent factors impacting the people's awareness rate of PSA, and the relations are statistically significant (*p* < 0.05; Table 4).

**Table 4:** Chi-square test and logistic regression analysis of PSA awareness in C-withME.

**Variables**	**Number**	**Chi-square test**		**Logistic regression analysis**		
		*X* ^2^	***P***	**OR**	**95% CI**	***P***
Education	Bachelor's degree or equivalent	175	85.25	< 0.001	-	-	-
	Graduate student and above	45			20.15	6.89–58.96	< 0.001
	engaged in the healthcare	90			9.83	5.20–18.58	< 0.001
Gender (male/female)		135/175	5.12	< 0.05	1.69	1.07–2.66	< 0.05
Age (< 30/≥30 years old)		187/123	69.34	< 0.001	9.75	5.45–17.43	< 0.001

### Cohorts with a medical educational background but without clinical practice (C-withoutCP)

There were 220 respondents in C-withoutCP. One hundred and twenty-five (56.8%) respondents in this group were female, and 95 (43.2%) were male. There was 81 people under the age of 20 (36.8%), 80 people (36.4%) aged 20–30 years, 36 people (16.4%) aged 30–40 years, and 23 people over 40 years old (10.4%). One hundred and seventy-five (79.5%) respondents were undergraduates or equivalent, and 45 (20.5%) were postgraduates and above. One hundred and three (46.8%) respondents were exposed to knowledge or cases related to PCa, and 117 (53.2%) were not. One hundred (45.5%) respondents were aware of PSA, and 120 (54.5%) were unaware.

For C-withoutCP, there were statistically significant differences between the AP group and the UAP group in terms of gender, age, degree, and whether they had been exposed to knowledge about PCa (*p* < 0.05; Table 5). Logistic regression analysis showed that males, age ≥ 30 years old, exposure to knowledge about PCa, and status of graduate student and above were the independent factors impacting people's awareness rate of PSA with statistically significant differences (*p* < 0.05; Table 5). In addition, the results of the logistic regression analysis showed that exposure to knowledge about PCa, the status of graduate students and above, and higher awareness of the clinical diagnosis stage of PCa were independent factors impacting people's future expectations of PSA with statistically significant differences (*p* < 0.05; Table 6).

**Table 5:** Chi-square test and logistic regression analysis of PSA awareness among C-withoutCP.

**Variables**	**Number**	**Chi-square test**		**Logistic regression analysis**		
		*X* ^2^	***P***	**OR**	**95% CI**	***P***
Gender (male/female)	95/125	5.810	0.016 < 0.05	0.515	0.300–0.886	< 0.05
Age (under 30/over 30 years old)	161/59	50.200	< 0.001	12.333	5.630–27.020	< 0.001
PCa knowledge (unexposed/exposed)	117/103	113.04	< 0.001	37.218	17.352–79.831	< 0.001
Degree (undergraduate or equivalent/graduate and above)	175/45	47.563	< 0.001	20.153	6.889–58.955	< 0.001

**Table 6:** Logistic regression analysis of PSA future expectations in C-withoutCP.

**Variables**	**B**	**S.E**.	**Wald χ^2^**	***P***	**OR**	**95% CI**		
Prostate cancer knowledge	Unexposed	-	-	-	-	-	-	-
	Exposed	1.171	0.293	16.007	< 0.001	3.226	1.818	5.726
Degree	Undergraduate or equivalent	-	-	-	-	-	-	-
	Graduate and above	1.337	0.418	10.202	< 0.01	3.806	1.676	8.643
Awareness of the stages at which PCa is diagnosed	Early stage	-	-	-	-	-	-	-
	Progress stage	1.269	0.467	7.368	< 0.01	3.557	1.423	8.892
Late stage	1.447	0.496	8.499	< 0.01	4.250	1.607	11.242

B, regression coefficient; S.E., standard error; Wald χ^2^, the Wald test statistic calculated from the data to be compared with the χ^2^ distribution with 1 degree of freedom.

### Cohorts with medical educational background and clinical practice (C-withCP)

In this study, 90 respondents were already engaged in healthcare. Among these 90 respondents, 50 were females (55.6%), and 40 were males (44.4%). Twenty-six (28.9%) were 20–30 years old, 35 (38.9%) were 30–40 years old, 15 (16.7%) were 40–50 years old, and 14 (15.6%) were over 50 years old. Thirty-five (38.9%) respondents jobs are related to urology, and 55 (61.1%) aren't. Fifty-two (57.8%) respondents had been exposed to PSA screening, and 75 (83.3%) respondents knew about PSA.

For C-withCP, there was no statistically significant difference between the AP group and the UAP group in terms of gender (*p* > 0.05; Table 7). However, there were statistically significant differences between the AP group and the UAP group in terms of different departments and whether they had been exposed to PSA screening (*p* < 0.05; Table 7). Furthermore, logistic regression analysis showed that departments related to urology and exposure to PSA screening were the independent factors impacting people's awareness rate of PSA with statistically significant differences (*p* < 0.05).

**Table 7:** Chi-square test and logistic regression analysis of PSA awareness among C-withCP.

**Variables**	**Number**	**Chi-square test**		**Logistic regression analysis**		
		*X* ^2^	***P***	**OR**	**95% CI**	***P***
Gender (male/female)	40/50	0.144	0.704 > 0.05			
Department (unrelated/relevant to urology)	55/35	7.864	0.005 < 0.05	11.610	1.452–92.843	< 0.05
Exposure to PSA screening (no/yes)	38/52	19.275	< 0.001	29.750	3.695–239.557	< 0.01

## Discussion

Early diagnosis and treatment of PCa can effectively improve the prognosis of the disease and patients' quality of life. Therefore, the research on PSA, a tumor marker for PCa, plays a crucial role in improving public health (5). Unfortunately, there have been very few studies on the awareness rate of PSA among Chinese residents in the past. In this study, we conducted a nationwide survey on the PSA awareness rate and analyzed the influencing factors with 493 samples. Although this study had some limitations, it still has a guiding significance for widespread scientific educational programs of PCa knowledge, which is representative and referential.

The results of this study indicate that the awareness rate of PSA among the general public in China is at a low level. Differences between the AP group and the UAP group in terms of age, educational backgrounds, occupation, degree, the habit of knowing medical knowledge, exposure to PCa patients, exposure to knowledge about PCa, and exposure to PSA screening vary significantly. However, there were no significant differences between the AP group and the UAP group regarding gender and whether they had heard of PCa. The awareness rate of PSA is higher among older people who are engaged in healthcare, who have the habit of understanding medical knowledge, who have been exposed to PCa patients, related knowledge or PSA screening, and who have a graduate degree or above. Older people who are engaged in healthcare, know PCa more comprehensively, and are aware of PSA prefer to learn about PSA. Age is the most influential factor in the PSA awareness rate and the PSA awareness rate is increasing significantly with age.

Among all respondents in this study, only 37.9% knew PSA and 62.1% did not. What's worse, the awareness rate of PSA among C-withoutME is only 6.5%, which is much lower than the overall awareness rate of PSA. It is prompted that health education on the prevention and treatment of PCa is still inadequate in China (14). A survey from Italy showed that the awareness rate of PSA among men in southern Italy is as high as 72.2% (15). A survey from Spain showed that the PSA awareness rate of the population reached 54.7%, and the PSA screening rate was 35.19% (16). However, the PSA awareness rate among C-withME is only 56.4% in China, which shows that even for C-withME, the PSA awareness rate is not optimistic. It suggests that there is still a significant gap in the PSA awareness rate between China and developed countries such as the United States. Moreover, there is a lack of scientific education on PCa for medical staff, and even more among non-medical personnel. Therefore, corresponding widespread scientific educational programs should be implemented soon.

Differences between the AP group and the UAP group in terms of age, educational backgrounds, occupation, degree, the habit of knowing medical knowledge, exposure to patients with PCa, exposure to PCa knowledge, and exposure to PSA screening vary significantly. C-withME have significantly more exposure to knowledge about prostate in their life than C-withoutME, so their PSA awareness rates are significantly higher. Older men account for the most significant proportion of PCa cases. Studies have shown that the incidence of PCa in China is low up to 60 but increases significantly and exponentially after 60, with a peak after 80 (9). The older the male is, the higher the risk of prostate cancer. PSA awareness increases with age due to more exposure to prostate knowledge, perhaps more so in men than women. However, surprisingly we found there was no significant difference between the AP group and the UAP group in terms of gender, which may be related to the fact that the age group of 493 samples was mainly focused on youth with a smaller sample size of middle-aged and older men, making it challenging to derive differential statistical analysis results. This survey showed no statistical difference between the AP group and the UAP group regarding whether they had heard of PCa, indicating that the science popularization of PCa is still at a superficial level to a certain extent. To achieve the goal of cancer prevention and treatment, increasing the breadth and depth of science popularization is necessary.

Older people who are engaged in healthcare, know prostate cancer more comprehensively and are aware of PSA, prefer to learn about PSA. The future expectation of PSA is higher with increasing awareness of PCa. Studies have shown that more knowledgeable people are more likely to be screened for prostate cancer (17). In this study, only 54.6% of people intended to know about PSA. In comparison, 36.5% are unaware and unwilling to know about PSA, suggesting that the future expectation of PSA among the population is at a low level as a whole, and the science popularization on prevention and treatment of PCa needs to be strengthened. The high number of people who are unaware and unwilling to know about PSA indicates that there is still a significant lack of health education nationwide, which needs to be strengthened in the future.

Studies have shown that the incidence rate and mortality of PCa in young men aged < 40 years increased significantly (9). Thus, it is worth noting that science popularization for young men aged < 40 is the top priority of future science popularization. Although the population with a high incidence of PCa is older men, the scientific popularization of prevention and treatment of PCa for young people and women still needs to be emphasized. After improving the awareness rate of PSA among young people, the PSA awareness rate of people at high risk can be improved through the popularization by young people to the elders in the family, which can increase effective disease screening. In China, family members often dominate the clinical decision-making process (18), which means that the implementation of patient medical measures is closely related to the decisions of family members. Due to their older age, lack of medical knowledge, and low level of clinical cognitive decision-making, PCa patients are more willing to negotiate with their families. Therefore, the younger generation makes most of the family's final clinical decisions (19). At the same time, studies have shown that women can effectively improve knowledge about preventing and treating PCa in men and play a positive role in men's screening intentions (20, 21). Moreover, women are more willing to participate in decision-making that can support and promote men's health (20, 21). Therefore, it can be seen that women and young people in the family play a key role in clinical decision-making. Improving the PSA awareness rate of women and the younger generation in the family can effectively promote the PSA awareness rate and screening rate of men at high risk. Therefore, it is necessary to include them in the study. In a nutshell, this study provided scientific data for science popularization among cohorts < 40 years old to promote PSA screening in cohorts at high risk.

Improving the PSA awareness rate is one of the keys to increasing the initiative of PSA screening in men at high risk. The survey results showed that effective health education on the prevention and treatment of PCa for men could significantly increase their awareness of PSA screening (17). Furthermore, effective science popularization is not only conducive to improving the awareness rate of different people about preventing and treating PCa, but also can improve the acceptance of PSA screening and its importance. This can achieve the early detection, diagnosis, and treatment of PCa, reducing the morbidity and mortality of PCa and improving the survival rate and quality of life for PCa patients.

Studies have shown that the existing health education textbooks in China support the current demand for health education knowledge among the population (22). Judging from the current situation in China, it is essential to carry out targeted, scientific, and systematic prostate knowledge science popularization for people with different academic levels, educational backgrounds, and ages. Therefore, scientific education targeting medical personnel, especially non-urologists, should be at the forefront. Furthermore, studies have shown that physicians are one of the essential sources of knowledge about PCa screening in the population (17), and a more significant proportion of those who have received PSA screening are recommended by physicians (15), it indicates physicians' recommendations are essential factors in promoting PSA screening behaviors (23). Thus, it is crucial for medical personnel, especially non-urologists, to master prostate cancer prevention knowledge and to publicize it to accessible men < 40 years old or at risk.

The majority targets of science popularization on the prevention and treatment of PCa should focus on cohorts < 40 years old in the future. With the development of the Internet era, platforms such as Microblogs and WeChat public numbers occupy a space in information diffusion and science popularization for various knowledge (24), in addition to short video platforms such as Auto Quicker and Tik Tok. Studies have shown that the primary way for the public to access health information is through online media (25). For cohorts < 40 years old, the main focus should be on online publicity, with equal emphasis on both extensive and in-depth popularization. At the same time, we should emphasize science popularization for the elderly.

The science popularization for the elderly should consider the particularity of the elderly group, with offline publicity as the primary and online publicity as the auxiliary. Facing different types of audiences, attention should be paid to health education with targeting.

Governments and medical agencies can take various measures to increase PSA awareness rate of the general public. First, governments and medical agencies should strengthen the training of professional personnel (25) so that more professionals can devote themselves to the publicity of prostate cancer prevention and treatment knowledge. In addition, governments and medical agencies can strengthen science popularization by building official online platforms. It is also feasible to strengthen the development of authoritative science popularization programs on TV (25). Due to the underdevelopment of the Internet and the difficulty of the elderly in using mobile devices proficiently, science popularization in rural areas should be based on offline publicity. Studies have shown that community-based health education measures, such as holding lectures and conducting symposiums, can effectively improve knowledge about cancer and cancer screening behaviors among the general public (22). Local charity organizations can organize professional science popularization volunteer service teams to conduct community publicity and science lectures. On top of that, free clinics can also be conducted periodically by medical agencies to provide science and health education to the elderly in the community and enhance health concepts.

There are still some limitations in this study. Firstly, this study is a cross-sectional survey, and the level of evidence is relatively low. Secondly, the sample size was small. Although the sample range covered several provinces in China, and the study results could reflect the PSA awareness rate among the general public, the proportion of individual provinces was large, and the data were unevenly distributed. Besides, we did not conduct a sample size design for sampling across the country, which may lead to selection bias. In addition, although the age of the samples in this study covered all age groups, we still had a more significant proportion of young adults and a smaller proportion of the elderly. The inability of older people to be proficient in using mobile devices to fill out questionnaires may be one of the reasons for this. However, this study aims to understand the PSA awareness rate of young family members and then provide scientific data for science popularization, thereby promoting PSA screening in men at high risk in their families. Online surveys may have participants casually answering questions due to reasons like participant disinterest or survey fatigue, which may affect the validity of the data. At the same time, online questionnaires cannot provide timely help to participants who need help understanding questionnaire questions, which is also a drawback of online questionnaire research.

## Conclusions

The PSA awareness rate in China is still low, with an even lower awareness rate among C-withoutME. The PSA awareness rate among medical personnel could be more optimistic. Age and educational backgrounds are the main factors impacting people's PSA awareness rate. The rapid increase in the incidence of PCa indicates that it is urgent to popularize the knowledge about PCa among the whole society. At the same time, it cannot be ignored that the science popularization goals should focus on cohorts < 40 years old in the future. In addition, we should build up well-established, robust, and community-based scientific educational programs on preventing and treating PCa with available resources for all ages and all people to improve effective screening of men at risk. When conducting the community-wide science popularization, the influencing factors of PSA awareness rate should be combined with the influencing factors of future expectations of PSA with careful consideration to achieve targeted and scientific popularization, enhance self-informed decision-making for PSA screening among men at risk, and improve educational programs on PCa prevention and treatment.

## Data availability statement

The original contributions presented in the study are included in the article/Supplementary material, further inquiries can be directed to the corresponding author.

## Ethics statement

The studies involving human participants were reviewed and approved by the Ethics Committee of Fujian Provincial Hospital. Written informed consent for participation was not required for this study in accordance with the national legislation and the institutional requirements.

## Author contributions

YWe: conceptualization. YWa, MX, YZ, ZH, RZ, QX, and LL: formal analysis and investigation. YWa, MX, and YZ: writing—original draft preparation. YWa and YWe: writing—review and editing. All authors read and approved the final manuscript.
